# Genetic Counseling in Renal Masses

**DOI:** 10.1155/2008/720840

**Published:** 2008-11-09

**Authors:** José Antonio López-Guerrero, Zaida García-Casado, Antonio Fernández-Serra, José Rubio-Briones

**Affiliations:** ^1^Laboratory of Molecular Biology, Fundación Instituto Valenciano de Oncología, C/Prof. Beltrán Báguena 8, 46009 Valencia, Spain; ^2^Service of Urology, Fundación Instituto Valenciano de Oncología, C/Prof. Beltrán Báguena 8, 46009 Valencia, Spain

## Abstract

All urologists have faced patients suffering a renal cancer asking for the occurrence of the disease in their offspring and very often the answer to this question has not been well founded from the scientific point of view, and only in few cases a familial segregation tree is performed. The grate shift seen in the detection of small renal masses and renal cancer in the last decades will prompt us to know the indications for familial studies, which and when are necessary, and probably to refer those patients with a suspected familial syndrome to specialized oncological centers where the appropriate molecular and familial studies could be done. Use of molecular genetic testing for early identification of at-risk family members improves diagnostic certainty and would reduce costly screening procedures in at-risk members who have not inherited disease-causing mutations. This review will focus on the molecular bases of familial syndromes associated with small renal masses and the indications of familial studies in at-risk family members.

## 1. INTRODUCTION

Renal cell carcinoma (RCC) affects approximately 150 000 people worldwide each year, causing close to 78 000 deaths annually, and its incidence seems to be rising [1]. This rising trend is partially due to the growing use of new and improved noninvasive abdominal imaging modalities, such as ultrasonography, CT, and MRI [2, 3]. In more recent years, 48–66% of RCCs have been detected incidentally as small renal masses in asymptomatic patients, whereas historically most cases were diagnosed following investigations for flank pain or hematuria [4]. RCC is not a single entity, but rather comprises the class of tumors of renal epithelial origin. Broad histological and molecular studies have resulted in a consensus classification of different RCC subtypes (Table 1) [5].

Most cases of RCC are thought to be sporadic whereas there has been estimated that hereditary RCC syndromes are estimated at 1–4% but have major clinical and scientific implications [6, 7]. First, the identification of predisposing gene offers the possibility of genetic testing: surveillance of mutation carriers results in early diagnosis and treatment. Secondly, the involvement of the same genes is demonstrated in a number of sporadic RCCs, providing insight into the various mechanisms of renal tumorigenesis [8]. To date, 10 familial syndromes associated with one or more of the various histological subtypes of RCC have been described, all of them inherited with an autosomal dominant trait, that means that carrier individuals of a mutant allele have a 50% chance of passing the mutant gene to the offspring and therefore the associated disorder (Table 2) [9]. The diverse nature of these predisposing genes implicates different mechanisms and biological pathways in RCC tumorigenesis. Hence, identification of mutations responsible for these syndromes in healthy carriers constitutes a challenge in the clinical management of these individuals.

There are no generally accepted screening guidelines for hereditary RCC syndromes; however, some recommendations can be made. A hereditary predisposition to renal cancer should be suspected whenever an individual who is diagnosed with renal cancer has a close relative also diagnosed with the disease, and/or when an individual presents with multifocal renal tumors or a history of previous renal tumor. Family history should be obtained and a pedigree created, paying specific attention to relatives with a known history of cancer. Whenever possible (when a gene-causing disease is identifiable), a germline genetic testing should be performed on the proband. In addition, and as a general rule, molecular genetic testing of at-risk family members is appropriate in order to identify the need for continued, lifelong, clinical surveillance. Interpretation of the result is most accurate when a disease-causing mutation has been identified in an affected family member. Those who have a disease-causing mutation require lifelong regular surveillance. Meanwhile, family members who have not inherited the mutation and their offspring have risks similar to the general population [10].

In this case, and generally speaking within a genetic testing context, the presence or absence of a mutation in a predisposing gene or the type of mutation determines the clinical actuation in cases of hereditary syndromes of cancer. In this sense, and following the American College of Medical Genetics (ACMG) recommendations, we can describe the following situations [10]:

**Situation 1.**

When the mutation is present:

- the pathogenic sequence alteration is reported in the literature;
- sequence alteration is predicted to be pathogenic but not reported in the literature;
- sequence variation of unknown clinical significance;
- sequence alteration is predicted to be benign but not reported in the literature;
- a benign sequence alteration is reported in the literature.

**Situation 2.**

Possibilities if a sequence alteration is not detected:

- patient does not have a mutation in the tested gene (e.g., a sequence alteration exists in another gene at another locus);
- patient has a sequence alteration that cannot be detected by sequence analysis (e.g., a large deletion, a splice site deletion);
- patient has a sequence alteration in a region of the gene (e.g., an intron or regulatory region) not covered by the laboratory's test.

Herein we review the four most frequent syndromes (von Hippel-Lindau, Hereditary papillary RCC, Hereditary leiomyomatosis RCC, and Birt-Hogg-Dubé), the molecular biology of the associated genes, and the clinical consequences of a genetic counseling.

## 2. VON HIPPEL-LINDAU (VHL) DISEASE

### 2.1. Clinical manifestation and molecular biology

VHL (OMIM: 193300) is the main cause of inherited RCC [11]. This syndrome includes central nervous system (CNS) and retinal hemangioblastomas, clear cell RCC and renal cysts, pheochromocytomas, neuroendocrine pancreatic tumors and pancreatic cysts, and endolymphatic sac tumors [12]. VHL occurs at a prevalence of about 1/36 000 and VHL-associated tumors with relatively high penetrance (80–90%) develop in the second to fourth decades of life. RCC affects up to 75% of patients by the age of 60 years. RCC is predominantly multiple and bilateral and occurs at a mean age of 39 years [11, 12] (Table 3).

Genetically, VHL is caused by germline mutations in the *VHL* tumor suppressor gene located on 3p25-26 accompanied by inactivation of the wild-type copy of the *VHL* gene in a susceptible cell through loss of heterozygosity (LOH), promoter hypermethylation, or somatic mutation [6].

VHL disease tumor suppressor protein (pVHL) has been implicated in a variety of functions including transcriptional regulation, posttranscriptional gene expression, protein folding, extracellular matrix formation, and ubiquitinylation [13]. The role of pVHL in the regulation of hypoxia-inducible genes through the targeted ubiquitinylation and degradation of hypoxia-inducible factor-1*α* (HIF1*α*) has been elucidated, leading to a model of how disruption of the *VHL* gene results in RCC and the production of highly vascularized tumors.

**Figure 1:**
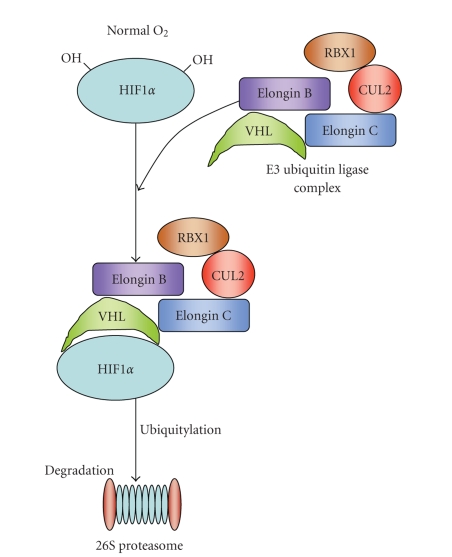
VHL complex interaction with HIF*α* under normal O_2_ levels. Its normal function leads to HIF*α* degradation (see text for details).

Under normoxic conditions, HIF1*α* is hydroxilated (−OH) on two conserved praline residues by a member of the EGLN family of prolyl hydroxylase enzymes. This hydroxylation provides a substrate-recognition site for the pVHL-E3 ubiquitin ligase complex, which contains elongins C and B, cullin-2 (CUL2), and RBX1. Polyubiquitylation of HIF1*α* by the VHL complex leads to its proteasomal degradation by the 26S proteasome [6] (Figure 1).

However, under hypoxic conditions, HIF1*α* is not hydroxylated, pVHL does not bind, and HIF1*α* subunits accumulate. HIF1*α* forms heterodimers with HIF1*β* and activates transcription of a variety of hypoxia-inducible genes (i.e., VEGF, EPO, TGF*α*, PDGF*β*). Likewise, when pVHL is absent or mutated, HIF1*α* subunits accumulate, resulting in cell proliferation and the neovascularization of tumors characteristic of VHL disease [13].

Mutations in the *VHL* gene either prevent its expression (i.e., deletions, and frameshifts, nonsense mutations, splice site mutations) or lead to the expression of an abnormal protein (i.e., missense mutations), and interesting genotype-phenotype correlations are emerging for VHL disease that relate to the development of RCC [14]. A group of *VHL* mutations termed type 1, comprising mostly deletions and premature-termination mutations that cause total loss of pVHL function, predispose to the entire spectrum of VHL-syndrome except pheocromocytomas [15]. By contrast, type 2 mutations, which are mostly missense changes that reduce pVHL activity, predispose to the entire VHL spectrum, including pheochromocytomas with or without RCC, called type 2B and type 2A, respectively [6]. Several studies have revealed that type 1 and type 2B mutations, which predispose to RCC, show complete loss of HIF1*α* ubiquitylation and regulation, whereas type 2A mutations result in an incomplete defect in HIF regulation [16]. However, type 2A mutations have been shown to disrupt binding of pVHL to microtubules and abrogate the associated microtubule-stabilizing function of pVHL, implicating defective cytoskeleton organization in this VHL phenotype [17]. A third VHL-syndrome subclass (type 2C) predisposes almost exclusively to pheochromocytomas [9]. Type 2C mutations produce pVHL that regulates HIF but is defective in fibronectin assembly, indicating a possible link between fibronectin-matrix assembly and pheochromocytoma development [17]. Another class of *VHL* point mutations inactivates pVHL function by disrupting proper protein folding mediated by chaperonin TriC/CCT [18]. More recently, two independent groups reported a reduced risk for RCC in individuals with a complete deletion of the *VHL* gene. This group of individuals would define a new VHL phenotype characterized by a low risk for both RCC and pheochromocytoma [19, 20].

### 2.2. Molecular genetic testing

The molecular genetic testing of *VHL* is mainly performed by sequence analysis of all three exons which detects point mutations and small deletions or insertions and that represents the 72% of *VHL* mutations, and deletion analysis (by means of Southern Blot, MLPA, quantitative PCR, etc.) for detecting partial or complete gene deletions, which account for approximately 28% of all *VHL* mutations [21, 22].

Over 300 different *VHL* germline mutations have been identified [6, 11]. The mutations occur in all three exons, with only a handful of mutations found in four or more families (i.e., delPhe76, Asn78Ser, Arg161X, Arg167Gln, Arg167Trp, Leu178Pro). Codon 167 is a *hot spot* mutation. A database of mutations in the *VHL* gene is maintained on the human gene mutation database website http://www.hgmd.cf.ac.uk/ac/index.php.

Molecular genetic testing is indicated in all individuals known to have or suspected of having VHL syndrome [23]. Since the detection rate for *VHL* gene mutations is nearly 100%, molecular testing may also be used to evaluate individuals with a single VHL-associated tumor and a negative family history of the disease. In addition, for individuals with manifestations of VHL syndrome who do not meet strict diagnostic criteria and who do not have a detectable *VHL* germline mutation, somatic mosaicism for a de novo *VHL* disease-causing mutation should be considered. In some instances, molecular genetic testing of the offspring of such individuals reveals a *VHL* mutation [24].

The level of mutation detection obtained by molecular genetic testing of the *VHL* makes it possible to effectively rule out VHL syndrome with a high degree of certainty in individuals with isolated hemangioblastoma, retinal angioma, or clear cell RCC, who have no detectable *VHL* disease-causing germline mutation; somatic mosaicism for a *VHL* gene mutation still needs to be considered in such individuals. A younger individual, especially one with multiple lesions, is more likely to have a germline *VHL* mutation than an older individual with a single lesion [25].

Since pheochromocytoma is part of the VHL syndrome spectrum and may occur as the exclusive manifestation of VHL syndrome (type 2C), individuals with a family history of these tumors, or those in whom the disease is bilateral or multifocal, should be offered molecular genetic testing for *VHL* germline mutations [26]. Germline *VHL* mutations are rare in simplex cases of unilateral pheochromocytoma (i.e., an affected individual with no family history of VHL syndrome), unless the individual is younger than age 20 years. Exceptions are those individuals with a family history that is more consistent with familial paragangliomas of the head and neck, which are caused by mutations in various subunits of the gene encoding succinic dehydrogenase *(SDH*) [27, 28], or those individuals who have features of other heritable diseases associated with pheochromocytoma such as multiple endocrine neoplasia type 2A or 2B or neurofibromatosis type 1 [25].

Use of molecular genetic testing for early identification of at-risk family members improves diagnostic certainty and reduces the need for costly screening procedures in those at-risk family members who have not inherited the disease-causing mutation [29]. In addition, the American Society of Clinical Oncologists (ASCO) identifies VHL syndrome as a Group 1 disorder, that is, a hereditary syndrome for which genetic testing is considered part of the standard management for at-risk family members [30]. Early recognition of manifestations of VHL syndrome may allow for timely intervention and improved outcome; thus, clinical surveillance of asymptomatic at-risk individuals, including children, for early manifestations of VHL syndrome is appropriate.

### 2.3. Genetic counseling

Genetic counseling is the process of providing individuals and families with information on the nature, inheritance, and implications of genetic disorders to help them make informed medical and personal decisions.

As mentioned above, VHL syndrome is inherited in an autosomal dominant manner, and we call proband (or index case) to the affected individual through whom a family with a genetic disorder is ascertained. It has been reported that about 80% of individuals diagnosed with VHL syndrome have an affected parent whereas de novo mutations of the *VHL* gene are estimated to occur in about 20% of probands. Recommendations for the evaluation of parents of a proband with an apparent de novo mutation include molecular genetic testing if the *VHL* disease-causing mutation in the proband is known. If the disease-causing *VHL* mutation in the proband is not known, ophthalmologic screening and abdominal ultrasound evaluation, at a minimum, should be offered to both parents [31].

In the case of the sibs of a proband, the risk of VHL syndrome to sibs depends upon the genetic status of the parents: if a parent of a proband is clinically affected or has a disease-causing VHL mutation, the sibs of the proband are at 50% risk of inheriting the altered gene; and if neither parent has the disease-causing VHL mutation identified in the proband, the sibs have a small risk of VHL syndrome because of the possibility of germline mosaicism in one parent (at present the incidence of mosaicism is not known) [24].

**Figure 2:**
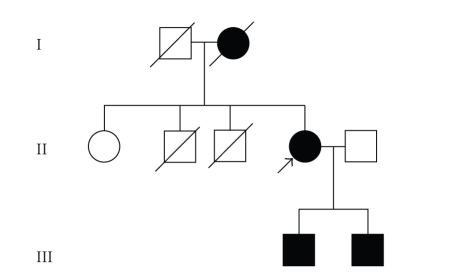
Pedigree showing affected members with VHL. *Open symbols*, unaffected subjects; *solid symbols*, affected subjects; *symbols with slashes*, deceased members; and *arrow*, proband.

Each offspring of an affected individual has a 50% risk of inheriting the mutant *VHL* gene; but the degree of clinical severity is not predictable (Figure 2), whereas the risk to other family members depends upon their biological relationship to the affected family member and can be determined by pedigree analysis and/or molecular genetic testing.

Molecular genetic testing of at-risk family members is appropriate in order to determine the need for continued clinical surveillance. Interpretation of molecular genetic test results is most accurate when a disease-causing germline mutation has been identified in an affected family member. Those who have the disease-causing mutation require regular surveillance, whereas family members who have not inherited the disease-causing mutation and their offspring need have no future concern [31].

Because early detection of at-risk individuals affects medical management, testing of asymptomatic individuals during childhood is beneficial [30]. As ophthalmologic screening for those at risk for VHL syndrome begins as early as possible, certainly before age five years, molecular genetic testing may be considered in young children. Molecular genetic testing may be performed earlier if the results would alter the medical management of the child.

The use of molecular genetic testing for determining the genetic status of presumably at-risk relatives when a family member with a clinical diagnosis of VHL syndrome is not available for testing is less straightforward. Such test results need to be interpreted with caution. A positive test result signals the presence of a VHL disease-causing mutation in the at-risk family member and indicates that the same molecular genetic testing method can be used to assess the genetic status of other at-risk family members. However, a negative test for a VHL gene mutation under such circumstances suggests one of the following possibilities:

- the at-risk family member has not inherited a *VHL* disease-causing mutation;
- the familial *VHL* mutation may not be detectable by the assays used; or
- the diagnosis of VHL syndrome in the affected family member is questionable.

In this situation, the presumably at-risk family member has a small, but finite, residual risk of having inherited a disease-causing allele (i.e., VHL syndrome or other hereditary disorder). In counseling such individuals, careful consideration should be given to the strength of the clinical diagnosis of VHL syndrome in the affected family member, the relationship of the at-risk individual to the affected family member, the perceived risk of an undetected *VHL* (or other) gene mutation, and the potential need for some form of continued clinical surveillance [31].

It is recommended that physicians ordering *VHL* molecular genetic testing and individuals considering undergoing testing understand the risks, benefits, and limitations of the testing prior to sending a sample to a laboratory. In fact, in some countries the individuals must give and sign an informed consent before the genetic analysis.

When neither parent of a proband with an autosomal dominant condition has the disease-causing mutation or clinical evidence of the disorder, it is likely that the proband has a de novo mutation. However, possible nonmedical explanations including alternate paternity or maternity (i.e., with assisted reproduction) or undisclosed adoption could also be carefully explored.

## 3. HEREDITARY PAPILLARY RCC

### 3.1. Clinical manifestation and molecular biology

Hereditary papillary RCC (HPRCC) (OMIM 605074) is characterized by the development of multifocal, bilateral papillary type-1 RCCs (low-grade tumors with basophilic cells and a favorable prognosis) occurring at a late age in ∼20% of gene carriers and a male/female ratio of 2:1 among affected members [6, 32] (Table 3). The pattern of inheritance is consistent with autosomal dominant transmission with reduced penetrance. Metastasis is less frequent, and age-dependent penetrance in mutation carriers seems to be reduced relative to penetrance in VHL syndrome [6].

**Figure 3:**
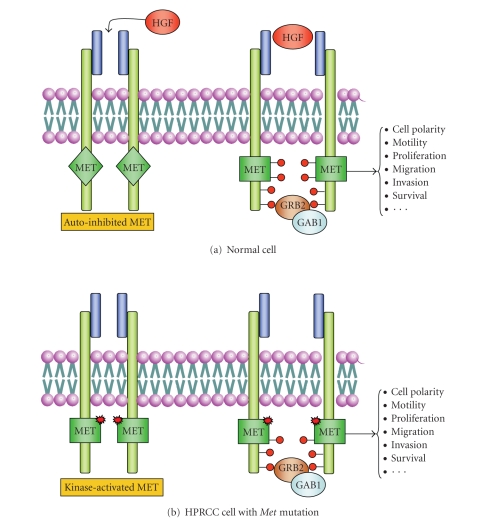
Activating mutations in MET in HPRCC. (a) In normal cells, hepatocyte growth factor (HGF) binds to MET receptor to induce MET dimerization and release autoinhibition. This permits, through several phosphorilation steps, the activation of second-messenger molecules (such as GRB2, GAB1, or PI3K) leading to morphogenic, motogenic, and mitogenic programmes. (b) Renal cells from patients with HPRCC can harbour germline mutations in the tyrosine kinase domain of MET. These mutations release the autoinhibition by the MET carboxyl terminus, allowing the transition of the receptor to the active kinase form in absence of ligand stimulation.

HPRCC is mainly caused by activating germline mutations in the tyrosine kinase domain of the *MET* proto-oncogene. *MET* is located in 7q31 and codifies a tyrosine kinase receptor that is normally activated by hepatocyte growth factor (HGF) [33] (Table 2). The MET–HGF signalling pathway is important for cell proliferation, epithelial–mesenchymal transitions, branching morphogenesis, differentiation and regulation of cell migration in many tissues. Most of the germline mutations occur within the MET activation loop or in the ATP-binding pocket and cause ligand-independent MET activation (Figure 3) [34].

Tumors from patients with papillary RCC and germline mutations of *MET* commonly show trisomy of chromosome 7 when analyzed by cytogenetic studies and comparative genomic hybridization (CGH) providing the second activating event in the renal cells [9].

### 3.2. Molecular genetic testing

The molecular genetic testing of *MET* is mainly performed by sequence analysis of exons 16 to 19. All reported alterations consist in point mutations. Ten known mutations are clustered in exons 16–19 of the tyrosine kinase domain and all are missense mutations which change the amino acid (V1110I, H1112R, H1112Y, M1149T, V1206L, V1238I, D1246N, Y1248C, Y1248D, M1268T). Mutations at four codons (V1110, D1246, Y1248, M1268) are homologous to sites of disease-associated activating mutations in other RTKs (RET, c-kit, c-erbB). Two unrelated North American families have been identified with the H1112R mutation and shared flanking genotyping data, suggesting a founder effect. Other mutations with only weak transforming potential (Y1248C, L1213V) confer anchorage-independent growth and an invasive phenotype in transfected cells.

Molecular genetic testing for a germline *MET* mutation is indicated in all individuals known to have or suspected of having HPRCC.

### 3.3. Genetic counseling

There are no specific screening guidelines for families suspected of having HPRCC. Individuals in these families are encouraged to talk with their doctor about screening options for kidney cancer, including ultrasound, and CT scan. Some clinicians suggest that individuals who have HPRCC, or a family history that suggests HPRCC, should have yearly screening beginning at age 30.

## 4. HEREDITARY LEIOMYOMATOSIS RCC

### 4.1. Clinical manifestation and molecular biology

Hereditary leiomyomatosis renal cell cancer (HLRCC) (OMIM 605839) predisposes to multiple cutaneous and uterine leiomyomas and solitary papillary type 2 RCCs [6, 35] (Table 2).

The majority of individuals (76%) present with a single or multiple cutaneous leiomyoma. These lesions appear as skin-colored to light brown papules or nodules distributed over the trunk and extremities, and occasionally on the face. Forty percent of individuals with HLRCC have mild cutaneous manifestations with five or fewer lesions [36]. Histologically, proliferation of interlacing bundles of smooth muscle fibers with centrally located long blunt-edged nuclei is observed.

Practically all females with HLRCC develop uterine leiomyomas [36–38]. However, whether all women with HLRCC have a higher risk of developing uterine leiomyosarcomas is unclear. In the original description of HLRCC, it was reported that 15% of women with uterine leiomyomas also had uterine leiomyosarcoma [39].

Most renal tumors are unilateral and solitary. Approximately 10%–16% of individuals with HLRCC who present with multiple cutaneous leiomyomas had renal tumors at the time that renal imaging was performed [37, 38]. Most tumors are classified as “type 2” papillary renal cancer, which display distinct papillary architecture and characteristic histopathology (high-grade tumors with large eosinophilic cells, an aggressive course, and a bad prognosis) [38] (Table 3). The median age at detection of renal tumors is 44 years, and, in contrast to other hereditary renal cancer syndromes, renal cancers associated with HLRCC are aggressive [38].

The disease is caused by germline mutations in the tumor suppressor gene *FH* located in 1q42-43 that encodes the mitochondrial Krebs cycle enzyme fumarate hydratase (EC 4.2.1.2.) [35]. *FH* consists of ten exons encompassing 22.15 kb of DNA and is highly conserved across species. The active form of the enzyme is a homotetramer and catalyzes the conversion of fumarate to L-malate. In mammals, there are two fumarase isoforms (mitochondrial and cytosolic) that are synthesized from the same mRNA. After initial synthesis, the FH proteins are partially imported and processed at the mitochondrial outer membrane [6].

Activity of FH enzyme can be measured in cultured skin fibroblasts or lymphoblastoid cells to confirm the diagnosis. Reduced activity (≤60%) of FH enzyme was found in all affected individuals with the diagnosis of HLRCC [40, 41].

The overall risk for renal tumor development is unclear and the mechanism of *FH*-mutation-driven tumorigenesis remains unknown so far [6]. It is plausible that intracellular fumarate accumulation as a result of *FH* inactivation causes decreased HIF degradation and overexpression of genes more downstream in the HIF pathway [42].

### 4.2. Molecular genetic testing

*FH* is the only gene known to be associated with HLRCC. Between 80% and 100% of individuals with HLRCC have identifiable sequence variants in *FH* [36–38]. The spectrum of mutations includes missense, insertion/deletion, and nonsense mutations that are predicted to truncate the protein, or substitute or delete highly conserved aminoacids, along with several whole-gene deletion. About 40 different *FH* mutations have been identified and are distributed throughout the entire gene without genotype-phenotype correlation [40]. Several of the mutations occur in many families, which could reflect a founder effect; notably, the Arg190His mutation, which is the most frequent mutation (33%) in a North American family study, and the Arg58X and Asn64Thr mutations in studies by the European-based Multiple Leiomyoma Consortium [6].

Molecular genetic testing for a germline *FH* mutation is indicated in all individuals known to have or suspected of having HLRCC, including individuals with the following:

- multiple cutaneous leiomyomas (with at least one histologically-confirmed leiomyoma) without a family history of HLRCC;
- a single cutaneous leiomyoma with family history of HLRCC;
- one or more tubulo-papillary, collecting-duct, or papillary type 2 renal tumors with or without a family history of HLRCC.

Measurement of FH enzyme activity can be useful in the diagnosis of HLRCC in cases with atypical presentation and undetectable *FH* mutations [40, 41].

No correlation is observed between *FH* mutations and the occurrence of cutaneous lesions, uterine fibroids, or renal cancer of HLRCC [36]. To date, six women with a germline mutation in *FH* have been reported with uterine leiomyosarcoma [43, 44]. It seems that *FH* mutation-positive families are in general not highly predisposed to uterine cancer, but a few individuals and families seem to be at high risk.

### 4.3. Genetic counseling

HLRCC is inherited in an autosomal dominant manner. Some individuals diagnosed with HLRCC have an affected parent and some have HLRCC as the result of a de novo gene mutation. In this case, the proportion of cases caused by de novo mutations is unknown as subtle manifestation in parents has not been evaluated and genetic testing data are insufficient. Recommendations for evaluation of parents of a proband with a suspected de novo mutation include molecular genetic testing if the *FH* disease-causing mutation in the proband has been identified. However, it is important to note that although some individuals diagnosed with HLRCC have an affected parent, the family history may appear to be negative because of failure to recognize the disorder in family members, early death of the parent before the onset of symptoms, or late onset of the disease in the affected parent.

In the case of the siblings of a proband, the risk depends upon the genetic status of the proband's parents. If a parent of a proband is clinically affected or has a disease-causing mutation, each sibling of the proband is at a 50% risk of inheriting the mutation. If the disease-causing mutation cannot be detected in the DNA of either parent, the risk to siblings is low, but greater than that of the general population because the possibility of germline mosaicism exists [38].

The risk to other family members depends upon the status of the proband's parents. If a parent is found to be affected or to have a disease-causing mutation, his or her family members are at risk.

It is not possible to predict whether symptoms will occur, or if they do, what the age of onset, severity, and type of symptoms, or rate of disease progression will be in individuals who have a disease-causing mutation.

When neither parent of a proband with an autosomal dominant condition has the disease-causing mutation or clinical evidence of the disorder, it is likely that the proband has a de novo mutation. However, possible nonmedical explanations including alternate paternity or undisclosed adoption could also be explored.

There is no consensus on clinical surveillance for HLRCC individuals so far but the following provisional recommendations have been accepted until a consensus conference is conducted [31].

Individuals with the clinical diagnosis of HLRCC, individuals with heterozygous mutations in FH without clinical manifestations, and at-risk family members who have not undergone molecular genetic testing should have the following regular surveillance by physicians familiar with the clinical manifestations of HLRCC.

- *Skin.* Full skin examination is recommended annually to every two years to assess the extent of disease and to evaluate for changes suggestive of leiomyosarcoma.
- *Uterus.* Annual gynecologic consultation is recommended to assess severity of uterine fibroids and to evaluate for changes suggestive of leiomyosarcoma.
- *Kidneys.* If both the initial (baseline) and the first annual follow-up abdominal CT scan with contrast are normal, this evaluation should be repeated every two years.

Any suspicious renal lesion (indeterminate lesion, questionable or complex cysts) at a previous examination should be followed with a CT scan with and without contrast. PET-CT may be added to identify metabolically active lesions suggesting possible malignant growth. It must be taken into consideration that ultrasound examination alone is never sufficient.

Renal tumors should be evaluated by a urologic oncology surgeon familiar with the renal cancer of HLRCC.

## 5. BIRT-HOGG-DUBÉ SYNDROME

### 5.1. Clinical manifestation and molecular biology

Birt-Hogg-Dubé (BHD) syndrome (OMIM 135150) is a genodermatosis that predisposes individuals to benign cutaneous lesions of the face and neck, spontaneous recurrent pneumothorax and/or lung cysts, and renal tumors [6, 7]. Approximately 15–29% of individuals with BHD syndrome have renal tumors [45, 46] (Table 3). The renal tumors are usually bilateral and multifocal. Tumor types include renal oncocytoma, chromophobe RCC, oncocytic hybrid tumor, and a minority of clear cell RCC [47]. The most common tumors are a hybrid of oncocytoma and chromophobe histologic cell types, so-called oncocytic hybrid tumor (67%), chromophobe RCC (23%), and renal oncocytoma (3%). Only renal oncocytoma is considered a benign tumor [48]. Other types of renal tumors reported in lower frequency include clear cell RCC and papillary renal carcinoma. Most renal tumors are slow-growing. Median age of diagnosis is 48 years with range from 31 to 71 years [46].

The disease is caused by germline mutations in the *BHD (FLCN)* gene on chromosome 17p11.2 [49]. *BHD* encodes folliculin, a new protein with unknown function but it is highly expressed in a variety of tissues including skin and skin appendages, type 1 pneumocytes, and distal nephrons of the kidney [50]. Recent studies suggest that folliculin might be involved in energy and/or nutrient sensing through the AMPK and mTOR signaling pathways [51].

*BHD* somatic mutations are very rare in sporadic RCC but hypermethylations are encountered in ∼30% of all RCC histological types [52]. Germline mutations in *BHD*, plus somatic mutations and loss of heterozygosity in tumor tissue, suggest that loss of function of the folliculin protein is the basis of tumor formation in BHD syndrome [53].

### 5.2. Molecular genetic testing

*BHD* is the only gene known to be associated with BHD syndrome. Various mutations have been identified in families with BHD syndrome. All mutations predict protein truncation. The most common mutation is cytosine insertion or deletion, which occurs in a polycytosine tract in exon 11, suggesting the presence of a hypermutable hot spot [46, 47]. Fifty-three percent of families with BHD syndrome have been found to have an insertion or deletion in the polycytosine tract in exon 11 (mutational hot spot) [46]. Sequence analysis of all coding exons (exon 4–14) increases the mutation detection in probands to 84% [46].

Molecular genetic testing is indicated in all individuals known to have or suspected of having BHD syndrome including individuals with the following.

1. Five or more facial or truncal papules with at least one histologically confirmed fibrofolliculoma [54] with or without family history of BHD.
2. A family history of BHD syndrome with a single fibrofolliculoma or a single renal tumor or history of spontaneous pneumothorax.
3. Multiple and bilateral chromophobe, oncocytic, and/or oncocytic hydrid renal tumors.
4. A single oncocytic, chromophobe, or oncocytic-hydrid tumor and a family history of renal cancer with any of the above renal cell tumor types.
5. A family history of autosomal dominant primary spontaneous pneumothorax without a history of chronic obstructive pulmonary disease.

Mutations in *BHD* were found in families with dominantly inherited spontaneous pneumothorax. Pulmonary involvement appears to be the only manifestation; penetrance is 100% [55, 56].

Acquired mutations in *BHD* have been identified in sporadic clear cell renal cell carcinoma [52, 57] and colon cancer [58, 59] without other associated tumors characteristic of the heritable disease.

No correlation is observed between type of *BHD* mutation and pulmonary and cutaneous manifestations. However, individuals who have a deletion in the polycytosine tract of exon 11 may have a lower risk of developing renal cancers than individuals with other mutations [46].

### 5.3. Genetic counseling

BHD syndrome is inherited in an autosomal dominant manner. Some individuals with BHD syndrome have an affected parent and some have BHD syndrome as a result of a de novo gene mutation. The proportion of cases caused by de novo mutations is unknown as a sufficient number of parents have not been evaluated for subtle manifestations, nor are there sufficient data on clinically unaffected parents who have been evaluated by molecular genetic testing. Recommendations for the evaluation of parents of a proband with a suspected de novo mutation include molecular genetic testing if the disease-causing mutation in the *BHD* gene in the proband is identified. But, although some individuals diagnosed with BHD syndrome have an affected parent, the family history may appear to be negative because of failure to recognize the disorder in family members, early death of the parent before the onset of symptoms, or late onset of the disease in the affected parent.

The risk to the siblings of the proband depends upon the genetic status of the proband's parents. If a parent of a proband is clinically affected or has a disease-causing mutation, the sibs of the proband are at a 50% risk of inheriting the mutation. If neither parent has the disease-causing mutation identified in the proband, the risk to sibs is low, but greater than that of the general population because the possibility of germline mosaicism exists.

When neither parent of a proband with an autosomal dominant condition has the disease-causing mutation or clinical evidence of the disorder, it is likely that the proband has a de novo mutation. However, other possible nonmedical explanations could also be explored.

There is no consensus on clinical surveillance; therefore, these recommendations are provisional until a consensus conference is conducted.

Individuals with known BHD syndrome, individuals known to have disease-causing mutations in *BHD* without clinical manifestation, and at-risk family members who have not undergone genetic testing should have regular monitoring by physicians familiar with the spectrum of BHD syndrome. In particular, surveillance for and monitoring of renal tumors include the following:

- if normal at baseline, abdominal/pelvic CT scan with contrast every two years;
- if any suspicious lesion (indeterminate lesion, questionable or complex cysts) at previous examination, annual abdominal/pelvic CT scan with contrast alternating every other year with MRI to reduce lifetime exposure to radiation;
- evaluation of renal tumors by a urologic surgeon;
- monitoring of tumors less than three centimeters in diameter by periodic imaging; they may not require surgical intervention while this small.

## 6. FUTURE TRENDS

The identification of genes responsible for inherited RCC has resulted in a better understanding of renal tumorigenesis including sporadic RCC and is paving the way for new therapeutic approaches [6, 7]. For VHL, recent and ongoing insights into the functions of the VHL gene, especially the HIF-ubiquitylation pathway, provide an attractive molecular basis for the development of specific inhibitors of HIF and/or its downstream targets [13]. Preliminary studies with the VEGF receptor inhibitor SU5416 showed that at least a third of patients with advanced VHL disease improved their clinical status giving promising expectations [60]. In same directions, new protein kinase receptor inhibitors are emerging [9]. In HPRCC, MET inhibitors gave encouraging results in in vitro studies, but clinical trials have started very recently and although data on the antitumor activity of the anti-MET compounds are not yet available, these studies have shown that MET inhibition results in low-grade toxicity, in agreement with the preclinical analyses performed in animal models [61, 62].

Recent studies suggest that HIF overexpression is involved in HLRCC tumorigenesis [42, 63]. Therefore, future target therapies for HLRCC-associated tumors may include, for example, anti-HIF therapies such as R59949 that regulate prolyl hydroxylase activity, thus preventing HIF accumulation.

The study of families with increased rates of cancer will continue to yield more insight into the factors that increase cancer risk. Genetic predisposition in the form of mutations and polymorphisms will increasingly be catalogued and DNA-level genetic profiling of high-risk families and individuals will become commonplace. The increase in availability of genetic testing and counseling for high-risk families should prove both helpful and cost-effective, as genetically unaffected family members reassured regarding their health status and removed from lifelong follow-up screening programmes.

Finally, we also should keep in mind, although not deeply discussed in this review, the psychological and ethical implications of the genetic counseling [64–66], not only from the strictly clinical point of view, but also regarding the management of personal genetic information that could have an impact on the individual and their relatives from certain health insurance companies.

## Figures and Tables

**Table 1:** Classification of renal epithelial tumors.

Histological type	Frequency	Cell of origin	Behavior	Gene involved	Chromosomal abnormalities
Conventional (clear-cell) renal-cell carcinoma	75%	Proximal renal tubule	Malignant	*VHL, BHD*	−3p, +5q, −Y, −8p, −9p, −14q;
					t(3;5)(p;q)
Papillary renal-cell carcinoma	10–15%	Proximal renal tubule	Malignant	*MET, FH, HRPT2*	+7, +17, −Y, +12, +16, +20;
					t(X;1)(p11.2;q21.2),
					t(X;17)(p11.2;q25.3)
Chromophobe renal carcinoma	5%	Intercalated cell of renal collecting duct	Rarely malignant	*BHD*	−1, −2, −6, −10, −13, −17, −21
Oncocytoma	5%	Intercalated cell of renal collecting duct	Benign	*BHD*	−1, −Y; t(5;11)(q35;q13),
					t(9;11)(p23;q13)
Collecting-duct carcinoma	2%	Renal collecting duct	Aggressively malignant	*FH*	−1p32, −6p, −8p, −21q

*BHD*, Birt-Hogg-Dubé (encoding folliculin); *FH*, fumarate hydratase; *HRTP2*, hyperparathyroidism 2; *VHL*, von Hippel-Lindau.

**Table 2:** Hereditary renal cell carcinoma (RCC) syndromes and histological subtypes.

Renal tumors	Manifestation	Disease	Gene
Clear cell RCC			
	Bilateral and multiple	Von Hippel-Lindau	*VHL*, 3p25-26
	Bilateral and multiple	Chromosome 3 translocations	Unknown, *VHL*?
		Hereditary paraganglioma	*SDHB*, 1p36
	Angiomyolipomas	Tuberous sclerosis	*TSC1*, 9q34
			*TSC2*, 16q13
Papillary RCC	Solid, bilateral and multiple (type 1)	Hereditary papillary RCC	*MET*, 7q31
	Unilateral solitary, aggressive (type 2)	Hereditary leiomyomatosis	FH, 1q42-43
	Hamartomas, Wilm's tumor	Hyperparathyroidism-jaw tumor	*HRPT2*, 1q25-32
	Oncocytoma	Familial papillary thyroid cancer	?, 1q21
Chromophobe RCC	Oncocytic-chromophobe	Birt-Hogg-Dubé	*BHD*, 17p11.2

**Table 3:** Hereditary patterns and risks of renal cell carcinoma (RCC) associated syndromes.

Syndrome	Hereditary pattern	Risk of developing an RCC of the affected individuals
*Von Hippel-Lindau*	Autosomal dominant	75%
*Papillary RCC*	Autosomal dominant	20%
*Leiomyomatosis RCC*	Autosomal dominant	10–16%
*Birt-Hogg-Dubé*	Autosomal dominant	15–29%
